# Menstrual health challenges in the workplace and consequences for women’s work and wellbeing: A cross-sectional survey in Mukono, Uganda

**DOI:** 10.1371/journal.pgph.0000589

**Published:** 2022-07-21

**Authors:** Julie Hennegan, Justine N. Bukenya, Fredrick E. Makumbi, Petranilla Nakamya, Natalie G. Exum, Kellogg J. Schwab, Simon P. S. Kibira

**Affiliations:** 1 Maternal, Child and Adolescent Health Program, Burnet Institute, Melbourne, VIC, Australia; 2 Department of Environmental Health and Engineering, The Water Institute, Johns Hopkins Bloomberg School of Public Health, Baltimore, MD, United States of America; 3 Department of Community Health and Behavioural Sciences, School of Public Health, College of Health Sciences, Makerere University, Kampala, Uganda; 4 Department of Epidemiology and Biostatistics, School of Public Health, College of Health Sciences, Makerere University, Kampala, Uganda; University of Murcia, SPAIN

## Abstract

This study describes women’s menstrual health needs at work in Uganda and explores the associations between unmet needs and women’s work and wellbeing. We undertook a cross-sectional survey of women working in marketplaces, public primary schools, and health care facilities in Mukono district, central Uganda. Survey questions were designed to capture women’s experiences of managing menstrual bleeding, pain, social support, and the social environment. A total 435 women working in markets, 45 teachers and 45 health care facility workers participated. Of these, 15% missed work due to their last period, and 41% would prefer not to work during menstruation. Unmet menstrual health needs were associated with consequences for women’s work and psychological wellbeing. Experiencing menstrual pain (aPR 3.65 95%CI 1.48–9.00), along with the use of improvised menstrual materials (aPR 1.41 95%CI 1.08–1.83), not feeling comfortable to discuss menstruation at work (aPR 1.54 95%CI 1.01–2.34) and the expectation that women should stay home when menstruating (aPR 2.44 95%CI 1.30–4.60) were associated with absenteeism due to menstruation. In contrast, not having menstrual management needs met (aPR 1.45 95%CI 1.17–1.79) and the attitude that menstruating women are dirty (aPR 1.94 95%CI 1.50–2.51), along with pain (aPR 1.59 95%CI 1.12–2.24) and norms around absenteeism were associated with wanting to miss work. After adjustment for age and poverty, unmet menstrual management needs (b = -5.97, 95%CI -8.89, -2.97), pain (b = -3.89, 95%CI -7.71, -0.08) and poor social support (b = -5.40, 95%CI -9.22, -1.57) were associated with lower wellbeing measured using the WHO-5. Attitudes that menstruation should be kept secret (b = 4.48, 95%CI 0.79, 8.17) and is dirty (b = 4.59, 95%CI 0.79, 8.40) were associated with higher wellbeing. Findings suggest that supporting care for menstrual pain, addressing secrecy surrounding menstruation and the perception of menstruation as dirty, and improving access to materials and facilities for managing menstrual bleeding are avenues for programs and policies to support working women.

## Introduction

Women’s participation in decent work is essential for sustainable development, reducing poverty, and improving the health of women and their families [1–4]. The many hours spent at work also makes workplaces important sites which can support, or undermine, health [5]. The average female menstruates 65 days of the year, yet women’s menstrual health needs in the workplace are frequently overlooked [6].

Menstrual health has been recognised as an essential part of sexual and reproductive health, and a core consideration for gender sensitive water, sanitation, and hygiene (WASH) service provision [7–11]. However, research to understand menstrual experiences and develop policy and practice responses in low- and-middle-income countries (LMICs) has focused almost exclusively on adolescent girls [12]. While adolescence represents a window of opportunity to safeguard menstrual health [13], menstrual health needs continue into adulthood [14,15]. Calls for greater attention to menstruation over the life-course have emphasised the need for research to understand women’s experiences at work and identify levers for improvement [6,16].

### Consequences of unmet menstrual health needs

Nationally representative surveys have found that many women report missing work or other daily activities due to menstruation. Performance Monitoring for Action surveys in Burkina Faso and Nigeria found 19% and 17% respectively, and almost one in four women in the lowest wealth tertile in both countries, missed work due to their period [15]. Multiple Indicator Cluster Surveys across countries have found up to 35% of women missed participating in school, work or other social events while menstruating [17]. While this nationally representative data highlights the importance of menstruation for women’s work, the surveys did not assess the reasons for absenteeism to inform policy and practice responses [18]. Further, a focus on attendance does not acknowledge other impacts on women’s lives [12,19,20]. Research with adolescent girls has been criticised for failing to capture impacts on participation at school, confidence or wellbeing, alongside attendance [21,22].

Prior qualitative interviews reported in Hennegan et al [14] undertaken with women working in markets, schools, and health care facilities in Mukono, Uganda found women experienced numerous consequences for their lives due to menstruation. These included: missed days at work, feeling uncomfortable or needing to persevere through distress or discomfort to remain at work, and anxiety and distress [14]. Thus, in this study we describe the prevalence of each of these consequences among our sample. Further, we investigate the extent to which different unmet menstrual health needs are associated with these outcomes. Women also reported implications of menstruation for their financial wellbeing, however this is outside the scope of this investigation.

### Unmet menstrual health needs

Past studies undertaken with adolescent girls have identified a wide range of unmet menstrual health needs across contexts [12]. These include: difficulties accessing sufficient materials to absorb or catch menstrual bleeding [20], poor availability of menstrual-friendly infrastructure to change materials or for cleaning the body and laundering materials [23], insufficient information about the menstrual cycle [24], inadequate support for reducing menstrual pain and discomforts [25], as well as social norms and attitudes surrounding menstruation which restrict behaviour and participation [12,20,26]. These challenges have been identified as core requirements for achieving menstrual health [7], and pillars for intervention design summarised briefly as: knowledge, materials and infrastructure for menstrual management, support for discomforts and disorders, and a supportive social environment [10]. Our interviews with women working in markets, schools and health care facilities echoed this body of evidence [14]. We found that strong social expectations to keep menstruation secret, and disgust surrounding menstruation resulting in strict hygiene requirements dictated women’s experiences. Menstrual pain and heavy bleeding were significant challenges, as was inadequately supportive infrastructure to change menstrual materials or wash the body. Difficulties affording sufficient menstrual materials for some, along with taboos held by market customers and varied social support from others at work also influenced women’s experiences of menstruation at work. In contrast to past research with adolescents [16], women working in markets, schools, and health care facilities did not emphasise menstrual knowledge gaps during our qualitative interviews [14].

Despite the breadth of menstrual health challenges identified in past qualitative research, there is scarce quantitative research to estimate the prevalence of unmet needs across different populations or to estimate the associations between these challenges and impacts on women’s lives [27]. Research that has been undertaken has often focused on the use of menstrual pads rather than the broad array of challenges identified through qualitative research [28,29]. Positively, more recent research with adolescents has investigated the contribution of a wider range of determinants including biological, menstrual management, and sociocultural considerations [30–32] but studies have not explored these associations in adult populations. It is plausible that different unmet menstrual health needs are more important contributors to different outcomes such as absenteeism or wellbeing. Understanding the associations between unmet menstrual health needs and consequences for women’s lives can help to prioritize program and policy responses and identify the outcomes that should be monitored in their evaluation.

### The present study

To address multiple gaps in the research evidence, the present study sought to address two aims. First, we aimed to describe working women’s unmet menstrual health needs and the prevalence of self-reported consequences for their work. Second, we aimed to test the associations between unmet menstrual health needs identified through our qualitative research and consequences for women’s lives, including (a) work absenteeism, (b) discomfort at work and (c) wellbeing, to inform future intervention approaches.

## Methods

This work is reported in accordance with the STROBE statement (included as S1 File) [33].

### Study population

Our research program included women working in public markets, government primary schools and public health care facilities (HCFs) in Mukono district, in the central region of Uganda. Mukono district was selected as an emerging industrial setting with a mix of rural and urban characteristics in which the study team had strong relationships with local governments and organizations. Market, school, and health care facility worker groups were selected as priority populations with government mandate for sanitation service provision [34]. Women working in marketplaces were the primary focus. In Uganda, informal employment has been estimated to account for 85% of non-agriculture employment [35], with many women in informal employment working as market vendors selling retail and food [36].

Markets operating in the district for at least 8 hours per day for 3 days per week were eligible. This excluded weekend-only markets, anticipating workers may have other week-day employment, and markets with restricted hours such as those only open for evening mealtimes. In collaboration with the local government, we identified 10 markets. Government primary schools and public HCFs in closest proximity to each market were then sampled. We recruited five teachers and five HCF workers for each of the 10 markets, sampled based on their availability with sampling extended to the next closest facility if there were less than five.

Women working in markets were sampled proportionally to the total population of female workers estimated from site visits and advice from market leaders. We sampled 50% of the population in each market, except for the largest market in the municipality which was under sampled (20%) to achieve sufficient representation from smaller markets. Enumerators mapped each market and systematically sampled female workers by selecting every second or fifth working woman. Women aged 18–45 who had worked at least 3 days per week over the past month and had not participated in the qualitative portion of the study were eligible. Ineligible women were replaced by the neighbouring female worker.

We sought a sample of 500 women in markets, alongside the 50 teachers and 50 HCF workers to explore sanitation needs and menstruation. The sample of teachers and HCF workers was limited by cost and feasibility. In our qualitative investigation [14] we found consistent consequences for women’s lives and the same set of contributing unmet menstrual health needs reported across worker groups. Thus, we hypothesised that associations between unmet menstrual health needs and consequences for work and wellbeing may be consistent across groups and all three groups were included in this quantitative study. Sensitivity analyses describe associations among the market sample alone. Our sample size was calculated to achieve 80% power to detect modest correlations between unmet needs and outcomes 0.20 (p<0.01) such as work absenteeism, while allowing up to 30% of the sample not to have had a menstrual period [37] and answer questions about menstrual health. Power calculations were undertaken prior to the qualitative study, and as such we did not specify a single outcome for these analyses, nor did we have any past research reporting on the prevalence of key variables such as discomfort at work or unmet menstrual health needs to draw on for this analysis.

### Data collection

Data were collected in March 2020. Surveys were programmed onto smartphones using Open Data Kit (ODK) and administered verbally, with data uploaded to a secure cloud server at the end of each day, and downloaded for analysis. Ten experienced female, Ugandan enumerators received five days of training on the survey tool, sampling, and informed consent process. Surveys were conducted in Luganda or English based on participant preference, with auditory privacy. Participants were informed of their right to decline to answer any questions and provided written informed consent for participation. Enumerators worked around participant schedules, pausing interviews for workplace tasks, or returning later as needed. Surveys lasted approximately 45–60 minutes, and participants received a bar of soap (approx. US$1) in appreciation.

### Measures

Survey questions were developed in English and designed to capture menstrual health needs, experiences, and consequences for women’s lives described across the integrated model of menstrual experience [12], with specific questions informed by past research [38,39] and findings from our qualitative investigation reported in Hennegan et al [14]. Questions were translated and back translated by bilingual research team members (JNB, SPSK, PN). Cognitive interviews with seven women were undertaken to assess question acceptability and comprehension, with modifications made as indicated. Questions were further workshopped during enumerator training. The full survey is available on the project page: www.osf.io/nzjtq. Broadly, survey topics included: demographic information, psychological wellbeing, life at work, biological menstrual characteristics such as pain, menstrual management practices and experience, the social environment, consequences for work and social participation, and women’s experiences of their workplace sanitation infrastructure.

#### Women’s work and wellbeing outcomes

We aimed to describe the prevalence of consequences for women’s work and test the associations between unmet menstrual health needs (outlined below) and three consequences for women’s lives identified through our qualitative study: (1) absence from work, (2) discomfort at work, and (3) wellbeing.

1. *Work absence*. Participants were asked to self-report if they usually missed work due to menstruation, and if they missed work due to their last period. Absence due to the last menstrual period was used as the outcome for multivariable analysis. We also asked how much time women missed and the reasons for absence for description.
2. *Discomfort at work*. Many women in qualitative interviews reported enduring significant discomfort or anxiety to remain at work during menstruation. In surveys women were asked: *“Would you avoid scheduling work (if possible) during your menstrual period*?*”*. This item was used in multivariable analysis to indicate those who are likely to experience discomfort at work due to menstruation.
3. *Wellbeing*. Psychological wellbeing was assessed through the widely validated World Health Organization Wellbeing Index (WHO-5) [40,41]. The measure focuses on recent wellbeing, with participants reporting how often over the past two weeks they experienced positive states such as feeling active and vigorous. Scores were calculated to range between 0 to 100 with higher scores representing greater wellbeing. In past studies, a score of ≤50 has been used as a screening diagnosis of depression [40].

#### Menstrual health needs (exposures)

Unmet menstrual health needs hypothesised to contribute to consequences for work and wellbeing were selected based on our qualitative findings reported in Hennegan et al. and grouped according to the categories identified through that analysis [14]. Table 1 displays the category reported in the qualitative study findings, identified unmet menstrual health need, and the survey question used. Further details on the measurement of each concept are provided below.

**Table 1:** Qualitative findings, identified unmet menstrual health needs, and corresponding survey measures used in the present study.

Category from qualitative findings [14]	Menstrual health need	Survey measure
Managing menses and cleaning the body	Menstrual management needs	Score on the Menstrual Practice Needs Scale.
	Use of improvised menstrual materials	The use of improvised materials (rather than commercial disposable or reusable pads) at work
Menstrual cycle characteristics	Pain	*“Do you experience cramping or pain in the abdomen*, *back or legs during your period*?*”*
	Pain severity	*“How would you rate the severity of your pain from 0–10*, *where 0 is no pain and 10 is the worst possible pain*?*”*
Workplace environment	Social support	*“How comfortable do/would you feel discussing menstruation with someone in your workplace*?*”* Very uncomfortable, uncomfortable, comfortable, very comfortable
	Social support	*“If your period started unexpectedly in the workplace*, *is there someone you could ask to help you*?*”* Agree/Disagree
Keeping clean	Supportive sociocultural environment	**Attitude**: *“Women should avoid working during menstruation for workplace hygiene”* Agree/Disagree
		**Injunctive norm: “***Women working here are expected to stay at home when they are menstruating”* Agree/Disagree
Keeping menstruation secret	Supportive sociocultural environment	**Attitude**: *“Women should not discuss menstruation with others in the workplace*, *it is a private matter”* Agree/Disagree
		**Injunctive norm: “***Most women working here expect others not to discuss menstruation”* Agree/Disagree
Modern knowledge and restrictions	Supportive sociocultural environment	**Injunctive norm**: *“Most people shopping in this marketplace would avoid purchasing food from a woman if they knew she was menstruating*.*”* Agree/Disagree

*Menstrual management needs*. Women’s experiences of managing menstrual bleeding were assessed through the Menstrual Practice Needs Scale (MPNS) [42]. This self-report scale assesses the extent to which women had access to resources and environments to care for their body which supported their preferences, comfort, privacy and safety during their last period [7]. Women reported whether needs were met on a four-point response scale from ‘never’ to ‘always’. The total mean score was calculated across all items applicable to the respondent. The MPNS performance in this population was assessed and is reported elsewhere [43]. The revised scale for adults was used for analysis. In contrast to past research, the scale was scored such that higher scores represent more negative experiences to support more easily interpreted prevalence ratios, with 0 representing the lowest level of unmet menstrual management needs, and 3 the highest. For description we also report differences across a categorical variable grouping respondents with a total score between 0 and 0.5 (few unmet needs), 0.51 and 1.49 (some unmet needs), and 1.5 to 2.49 (many unmet needs).

*Use of improvised menstrual materials*. The use of improvised materials (rather than commercial disposable or reusable pads) at work was included as a dichotomous independent variable. For 10 women who reported not attending any work during the last menstrual period, their menstrual material used at home was incorporated to avoid missing data.

*Pain*. Women were asked to report if they experienced menstrual pain and those experiencing pain were asked to rate the usual severity from 0 to 10. Those experiencing no pain were considered to have a severity of ‘0’.

*Social support*. Support in the workplace was assessed through two items, reported in Table 1, assessing women’s comfort discussing menstruation with someone at work and access to someone who could help them if their period started unexpectedly. Women’s comfort discussing menstruation with someone in their workplace was dichotomized as “uncomfortable” and “comfortable”.

*Sociocultural environment*. In qualitative interviews (see Table 1) women’s attitudes and the norm that menstruation should be kept secret, and that menstruation was dirty, so women needed to ‘keep clean’ were key determinants of menstrual experiences. In our survey we assessed women’s own endorsement of this attitude, the presence of the corresponding descriptive norm (what the respondent believes others do) and injunctive norm (what the respondent believes women are expected to do) [44]. Our qualitative findings highlighted the importance of the expectations of others, thus the injunctive norm was included for quantitative analysis. Women working in markets were also asked about customer behaviour highlighted during qualitative interviews, reporting if they felt shoppers would avoid menstruating women. Responses were included in sensitivity analysis undertaken with only women working in markets.

#### Demographic characteristics

Questions captured respondents’ age, marital status, level of education and other workplace details such as job type and days worked. Poverty was assessed through a 5-item lived poverty index [45] which asked how often over the past year the participants’ household had gone without food, water, medical treatment, fuel for cooking or cash income. A total score (0–20) was calculated.

The practices undertaken to manage menstrual bleeding, such as the type of absorbent used and disposal mechanisms were captured using questions from the Menstrual Practices Questionnaire to describe the sample [46].

### Analysis

Analyses were conducted using Stata 17. To address the first study aim we use descriptive statistics to report the prevalence of unmet menstrual health needs, and the prevalence of consequences for women’s lives including self-reported absenteeism, discomfort at work, and wellbeing.

To assess the associations between unmet menstrual health needs and absenteeism, discomfort at work and wellbeing (Aim 2) we tested the bivariate and multivariable associations between menstrual health needs and these outcomes. For dichotomous work consequences (absenteeism and discomfort) we used Poisson regressions with a robust variance estimator to provide prevalence ratios [47]. This method was selected as neither outcome was rare and thus odds ratios would represent a poor approximation of risk ratios [48]. To account for clustering at the level of the workplace we used generalized estimating equations with exchangeable correlation structure (assuming observations within the cluster are equally correlated) to provide a population-averaged effect [49]. Due to the small number of clusters (n = 29) we computed bias-corrected standard errors using the Kauermann and Carroll correction for the full sample [50]. Needs associated with consequences at p < .10 in bivariate analyses were included in the multivariable comparisons.

To test the associations between unmet menstrual health needs and wellbeing we undertook ordinary least-squares regression, with standard errors adjusted for clustering within workplaces. As generalised wellbeing was assessed, we adjusted for demographic factors (age and poverty) to assess the association between each menstrual health need and wellbeing individually (model 1). Associations with p < .10 were carried through to a multivariable model (model 2) to assess the relative contribution of different menstrual health needs.

Given the limited number of teachers and HCF workers included in the study, we undertook sensitivity analysis to explore the associations reported exclusively among women working in markets.

### Ethical approvals

Ethical approval was provided by Makerere University School of Public Health Higher Degrees, Research and Ethics Committee (HDREC: 739) and Johns Hopkins Bloomberg School of Public Health Institutional Review Board (IRB: 00010015). The Uganda National Council for Science and Technology (UNCST) approved the study (ref: SS 5143). Workplace administrators (Headteachers, Health Care Facility Administrators and Market Chairpersons) permitted recruitment of participants from their workplaces. Approval for the study in the area was also provided by the Mukono district chief administrator’s office and the Mukono Municipality Town clerk’s office.

## Results

### Respondents

Of the 600 women who participated in the quantitative survey, 87.5% had menstruated in the past six months and were asked questions about their menstrual experiences (n = 525). A total of 435 women working in markets, 45 teachers, and 45 HCF workers are thus included in this study.

Table 2 describes the characteristics of the sample. A total 83.0% of the sample reported having gone without food, water, fuel, medicines, or income within the past year. The mean and median days worked was 6, and almost half the sample (42%) worked 7 days per week. Most of the sample spent 9 to 12 hours in the workplace on a typical workday. Of those working in markets, 70.3% selected their own market hours, with a further 14.7% reporting that hours were dictated by the number of customers. Just 14.0% had a supervisor who determined their work hours.

**Table 2:** Sample characteristics.

	n	%
**Age**		
18–25	155	29.5
26–30	139	26.5
31–35	77	14.7
36–40	95	18.1
41–45	59	11.2
**Religion**		
Christian	424	80.8
Muslim	101	19.2
**Highest education level attended**		
None or primary school	188	35.8
Secondary school	244	46.5
Post-secondary school	93	17.7
**Usual days worked**		
3–4	74	14.1
5	83	15.8
6	150	28.6
7	218	41.5
**Hours worked in a typical day**		
<9	61	11.6
9–12	313	63.4
13+	151	28.8
**Menstrual material used most often at work during the last period (n = 514)**		
Cloth	85	16.5
Disposable pad	363	70.6
Reusable pad	42	8.2
Other: toilet paper, cotton wool or underwear alone	24	4.7
**Washed and reused any materials during the last period**		
Yes	145	27.6
No	380	72.4
**Changed menstrual materials at work during the last period**		
Never	28	5.3
One or some days	142	27.1
Every day	354	67.6

### Consequences for work and wellbeing

Table 3 displays the prevalence of work consequences due to menstruation and wellbeing reported for each worker group. A total of 19.3% of respondents reported usually missing work due to their period, 15.1% due to the last menstrual period, and 40.6% reported they would avoid scheduling work, if possible, during their period. Pain was the most common reason reported for absenteeism, along with other physical symptoms such as fatigue. A total 43% of those missing work included concerns about menstrual management or facilities as a reason for absenteeism.

**Table 3:** Prevalence of self-reported consequences for women’s work and wellbeing.

	Markets n = 435 n (%)	HCF n = 45 n (%)	Teachers n = 45 n (%)
**Work**			
Usually misses work due to menstruation	94 (21.7)	6 (13.3)	1 (2.2)
Missed work due to last menstrual period	72 (16.6)	6 (13.3)	1 (2.2)
Time missed during the last menstrual period (n = 79)			
< 1 day	6 (8.3)	0	0
1 day	31 (43.1)	4 (66.7)	1 (100.0)
2 days	18 (25.0)	0	0
3+ days	23.6 (17)	2 (33.3)	0
Reasons for missing work (n = 79)^1^			
Pain	54 (75.0)	5 (83.3)	1 (100.0)
Other physical symptoms: fatigue, heavy bleeding, gastrological symptoms	17 (23.6)	1 (16.7)	0
Containment fears or inadequate materials	27 (37.5)	3 (50.0)	0
Inadequate sanitation facilities	4 (5.6)	1 (16.7)	0
Other	2 (2.8)	0	0
**Discomfort at work**			
Would prefer not to work during menstruation	199 (45.8)	9 (20.0)	5 (11.1)
**Wellbeing**	**Mean (SD)**	**Mean (SD)**	**Mean (SD)**
WHO5	47.1 (22.06)	51.75 (20.03)	50.93 (19.68)

^1^ multiple response options, presents % of cases, does not add to 100.

### Associations between menstrual health needs and consequences for work

Table 4 displays the total prevalence of menstrual health needs among the sample. Scores on the MPNS ranged from 0 to 1.89 with a mean of 0.53 (SD = 0.40). Most of the sample reported few unmet needs (56.6%), with 41.3% reporting some unmet needs and 2.1% many unmet needs. Most women reported experiencing menstrual pain. Half of participants reported they would feel comfortable discussing menstruation with someone in the workplace, and 46.6% had someone they could ask for help. Over half of the sample believed menstruation should be kept secret, a greater 68.4% reported perceiving a norm that women should not discuss menstruation, and 15.6% agreed that women were expected to stay home from work while menstruating.

**Table 4:** Prevalence of menstrual health needs, bivariate and multivariable associations between reported menstrual health needs and work absenteeism and discomfort in Mukono, Uganda.

	Total % (mean)	Missed work n (%) / M (SD)	Did not miss work n (%) / M (SD)	PR (95%CI)	aPR (95%CI)	Would prefer to miss work n (%)	Would not prefer to miss n (%)	PR (95%CI)	aPR (95%CI)
Poverty	(4.33)	5.48 (3.95)	4.11 (3.66)	**1.07 (0.99–1.17)**	1.01 (0.92–1.10)	5.04 (3.95)	3.85 (3.51)	**1.04 (1.02–1.07)**	1.00 (0.99–1.02)
**Managing menses**									
Menstrual Practice Needs total score (0–3)	(0.53)	0.67 (0.47)	0.51 (0.38)	**2.00 (1.25–3.19)**	1.24 (0.85–1.79)	0.64 (0.43)	0.45 (0.36)	**1.85 (1.37–2.50)**	**1.45 (1.17–1.79)**
Few unmet needs	56.6	40 (13.5)	257 (86.5)			102 (34.3)	195 (65.7)		
Some unmet needs	41.3	35 (16.2)	181 (83.8)			104 (47.9)	113 (52.1)		
Many unmet needs	2.1	4 (36.4)	7 (63.6)			7 (63.6)	4 (36.4)		
Uses improvised materials at work (yes)	21.8	26 (22.8)	88 (77.2)	**1.67 (1.30–2.16)**	**1.41 (1.08–1.83)**	57 (50.0)	57 (50.0)	1.18 (0.85–1.66)	
Uses commercial materials at work	78.2	52 (12.7)	357 (87.3)	1.00		155 (37.8)	255 (62.2)	1.00	
**Pain**									
Experiences pain (yes)	77.7	74 (18.2)	333 (81.8)	**3.97 (0.98–16.16)**	**3.65 (1.48–9.00)**	184 (45.1)	224 (54.9)	**1.71 (1.21–2.42)**	**1.59 (1.12–2.24)**
Does not experience pain	22.3	5 (4.3)	112 (95.7)	1.00	1.00	29 (24.8)	88 (75.2)	1.00	1.00
Pain severity (1–10)	(4.45)	5.97 (2.86)	4.18 (3.36)	**1.15 (1.09–1.21)**	-	5.30 (3.27)	3.87 (3.28)	**1.07 (1.03–1.12)**	-
**Social support at work**									
Comfortable talking to someone (yes)	50.0	30 (11.5)	232 (88.6)	1.00	1.00	89 (33.8)	174 (66.2)	1.00	1.00
Not comfortable talking to someone	50.0	49 (18.7)	213 (81.3)	**1.59 (1.12–2.26)**	**1.54 (1.01–2.34)**	124 (47.3)	138 (52.7)	**1.36 (1.09–1.69)**	1.19 (0.99–1.43)
Has someone she could ask for help (yes)	46.6	40 (16.4)	204 (83.6)	1.00	-	93 (38.0)	152 (62.0)	1.00	-
Does not have someone	53.4	39 (13.9)	241 (86.1)	0.84 (0.52–1.36)	-	120 (42.9)	160 (57.1)	1.04 (0.90–1.20)	-
**Sociocultural environment: Attitudes & norms**					-				
Attitude: Menstruation should be kept secret									
Agree	56.8	49 (16.5)	248 (83.5)	1.22 (0.58–2.56)	-	140 (47.1)	157 (52.9)	**1.35 (1.13–1.62)**	1.11 (0.98–1.26)
Disagree	43.2	30 (13.3)	196 (86.7)	1.00	-	73 (32.2)	154 (67.8)	1.00	1.00
Attitude: Women should avoid work during menstruation for hygiene									
Agree	33.5	43 (24.6)	132 (75.4)	**2.31 (1.48–3.61)**	1.56 (0.90–2.69)	115 (65.3)	61 (34.7)	**2.33 (1.74–3.13)**	**1.94 (1.50–2.51)**
Disagree	66.5	36 (10.4)	331 (89.6)	1.00	1.00	97 (28.0)	250 (72.0)	1.00	1.00
Injunctive norm: Women are expected to keep menstruation secret									
Agree	68.4	61 (17.2)	294 (82.8)	**1.66 (1.01–2.71)**	1.08 (0.71–1.66)	161 (45.2)	195 (54.8)	**1.45 (0.95–2.22)**	1.10 (0.63–1.94)
Disagree	31.6	17 (10.4)	147 (89.6)	1.00	1.00	47 (28.7)	117 (71.3)	1.00	1.00
Injunctive norm: Women are expected to stay home when menstruating									
Agree	15.6	28 (34.6)	65.43 (53)	**3.02 (2.13–4.30)**	**2.44 (1.30–4.60)**	57 (69.5)	25 (30.5)	**1.97 (1.52–2.55)**	**1.49 (1.02–2.18)**
Disagree	84.5	49 (11.1)	391 (88.9)	1.00	1.00	153 (34.8)	287 (65.2)	1.00	1.00

PR: Prevalence Ratio. aPR: Adjusted prevalence ratio. CI: Confidence Interval.

Table 4 also presents women’s self-reported work absenteeism and discomfort according to unmet menstrual health needs, and the bivariate and multivariable associations between these unmet needs and work consequences.

*Absenteeism*. In the multivariable model, experiencing pain was associated with a much greater prevalence of absenteeism. Using an improvised (rather than commercially produced) menstrual material was associated with a greater prevalence of missing work. Not feeling comfortable to discuss menstruation and believing that women are expected to stay home during menstruation was also associated with absenteeism. The level of pain (rated from 0 to 10) was associated with absenteeism in bivariate comparisons, but due to high collinearity with reporting pain and 0-inflation we used the experience of pain as the predictor variable in the multivariable model.

Sensitivity analysis undertaken with only market women are reported in S2 File. The pattern of results remained the same as for the full sample. Agreeing that shoppers would avoid purchasing from a menstruating woman was associated with absenteeism in the bivariate comparison (PR = 1.50, 95%CI 1.01–2.19) but no longer statistically significant in the multivariable analysis (PR = 1.17, 95%CI 0.77–1.77).

*Discomfort at work*. Menstrual practice needs were significantly associated with wanting to miss work during menstruation, with an increase in 1 point on the MPNS associated with 1.45 times higher prevalence of wanting to avoid work during menstruation. Use of improvised materials was not associated with the desire to miss work. Pain remained associated, but social support was no longer statistically significant. Agreeing that women should avoid work during menstruation for hygiene was associated with preferring not to work during menstruation, along with the norm that women are expected to stay home. The attitude and norm that menstruation should be kept secret were associated with discomfort in bivariate comparisons but not in the final multivariable model.

In sensitivity analysis with market women, the pattern of results was similar, however pain was no longer significantly associated with wanting to miss work in the multivariable model (aPR = 1.36 95%CI 0.99–1.94), although this broader confidence interval may request the reduced sample. Not feeling comfortable to talk to someone at work was associated with a higher prevalence of desiring to miss work (aPR = 1.15 95%CI 1.02–1.30) with a similar effect size to that reported for the full sample (aPR = 1.19). For market women, believing that shoppers would avoid purchases from a menstruating woman was associated with wanting to miss work in the multivariable model (aPR = 1.39 95%CI 1.07–1.80).

### Associations between menstrual health needs and wellbeing

Both age and poverty were significantly associated with wellbeing scores and were included as covariates in assessing the multivariable associations between menstrual health needs and wellbeing (see Table 5). Having unmet menstrual practice needs was associated with poorer wellbeing in the individual adjusted model (model 1) and the full multivariable model with a 1-point increase on the MPNS associated with a 6-point decrease in wellbeing (WHO-5) score. The use of improvised materials at work was not significantly associated with wellbeing.

**Table 5:** Associations between reported menstrual health needs and wellbeing measured using the WHO-5.

	Model 1 (predictor with adjustment for age & poverty)		Model 2 (full multivariable model) (n = 520)	
Predictor	b (Std. Error)	95%CI	b (Std. Error)	95%CI
Age	**-0.35 (0.13)**	-0.63, -0.08	**-0.34 (0.13)**	-0.59, -0.08
Poverty	**-1.93 (0.22)**	-2.38, -1.47	**-1.75 (0.22)**	-2.20, -1.30
**Managing menses**				
Menstrual Practice Needs total score	**-7.50 (1.38)**	-10.33, -4.66	**-5.97 (1.47)**	-8.98, -2.97
Uses improvised materials at work (Yes)	1.96 (1.48)	-1.06, 4.99	-	
**Pain**				
Experiences pain (Yes)	**-4.51 (1.92)**	-8.45, -0.56	**-3.89 (1.86)**	-7.71, -0.08
Pain severity (1–10)	-0.53 (0.28)	-1.10, 0.05	-	
**Social support at work**				
Comfortable talking to someone (No)	**-5.02 (1.66)**	-8.42, -1.62	**-5.40 (1.87)**	-9.22, -1.57
Has someone she could ask for help (No)	-2.15 (2.14)	-6.54, 2.24	-	
**Attitudes & norms**				
Attitude: Menstruation should be kept secret (Agree)	3.13 (1.58)	-0.12, 6.38	**4.48 (1.80)**	0.79, 8.17
Attitude: Women should avoid work during menstruation for hygiene (Agree)	**4.55 (1.81)**	0.84, 8.26	**4.59 (1.86)**	0.79, 8.40
Injunctive norm: Women are expected to keep menstruation secret (Agree)	-1.18 (1.65)	-4.57, 2.21	-	
Injunctive norm: Women are expected to stay home when menstruating (Agree)	**4.12 (1.30)**	1.45, 6.79	1.79 (1.33)	-0.93, 4.51
Intercept			70.27 (5.15)	
Adj R^2^			0.18	

b: Regression coefficient. Std. Error: Robust standard error with cluster adjustment.

Experiencing pain during menstruation was associated with lower wellbeing as was not being comfortable to talk to someone at work about menstruation, with results presented in Table 5. Endorsing attitudes that menstruation should be kept secret, and that work should be avoided for workplace hygiene, suggesting a view of menstruation as dirty, was associated with greater wellbeing in the full multivariable model. Believing that others expected secrecy, or women to remain at home during menstruation, were not associated with wellbeing in the multivariable model. Together, age, poverty and menstrual health needs accounted for 18% of the variance in WHO-5 index scores.

The pattern of results was broadly consistent for sensitivity analysis including only women working in markets (see S2 File), however for this group pain was not significantly associated with wellbeing in the individual model (model 1). Wide confidence intervals meant attitudes that menstruation should be kept secret and that women should avoid work were no longer statistically significant in this more restricted sample (*p* = 0.067 and 0.090, respectively). Reporting that shoppers would avoid purchases from a menstruating woman was not significantly associated with wellbeing in the individual adjusted model (model 1).

## Discussion

Our study aimed to (1) describe the menstrual health needs and menstrual-related consequences for women’s lives in Mukono, Uganda, and (2) explore the associations between unmet menstrual health needs and consequences for work and wellbeing. We found a high prevalence of absenteeism and discomfort due to menstruation. Three in every 20 participants reported missing work due to their last period, and two in five reported that they would avoid scheduling work during menstruation if it were possible. Women reported many unmet menstrual health needs, including challenges with pain and caring for their body during menstruation, and difficulties in receiving social support and the social environment. In exploring the associations between these needs and work and wellbeing outcomes, we found that pain and social support were associated with absenteeism, while difficulties managing menstrual bleeding and social attitudes surrounding menstrual hygiene were associated with discomfort at work. Our findings provide the first quantitative evidence that menstrual health challenges may negatively impact adult women’s mental health. Results highlight that support for pain management, materials, and facilities for managing menstrual bleeding, and addressing stigma and silence surrounding menstruation are all important avenues for intervention. Addressing different unmet menstrual health needs may help to alleviate different consequences, with pain particularly critical for absenteeism and menstrual management essential for supporting comfort at work.

Consistent with research among adolescents [51], and surveys from high-income countries [52,53], menstrual pain was associated with work absenteeism. Of those who missed work, three quarters mentioned pain as a reason for absenteeism. Of those experiencing pain during menstruation, 18% missed work because of their last period, compared to 4% who did not report pain. Counter to hypotheses, reporting unmet menstrual management needs was not associated with absenteeism in multivariable comparisons despite many of those who missed work reporting management concerns as one of their reasons. However, use of an improvised menstrual material such as reusable cloth was associated with higher absenteeism. This was consistent with studies testing the associations between pad use and absenteeism that did not include other menstrual health needs [29]. Women reported varied menstrual material preferences in our qualitative interviews, but these quantitative results suggest that improvised materials may not perform as well as commercial products. It is possible that a greater risk of leakage or more time required to change these materials contributed to absences [54]. Not feeling comfortable to talk to someone at work about menstruation was associated with a higher prevalence of absenteeism, although reporting having someone to ask for help was not. This may suggest that the degree of comfort more validly captured women’s openness about menstruation and the support received. The expectation that women should stay home when menstruating was associated with a higher prevalence of absenteeism. This may reflect a negative expectation that menstruating women should not be present at work but could also capture those receiving greater permission from supervisors or co-workers to stay home if needed. In qualitative interviews many women reported that their supervisor would not be supportive if they needed to miss work due to menstruation [14], so it is possible this may be viewed as a positive norm.

This study advances evidence on the impacts of unmet menstrual health needs by exploring consequences for discomfort and wellbeing, not only absenteeism. Many women in our study reported desiring to miss work, indicating discomfort. Having unmet menstrual management needs was an important predictor in binary and multivariable comparisons. A total 48% of women with some unmet management needs reported they would rather miss work, compared to 34% of those with few unmet needs. Reflecting our qualitative finding that menstruating women were viewed as dirty, the attitude that women should avoid work during menstruation for hygiene, and the social expectation that women should stay home during menses was associated with the desire to miss work. A total 65% of women who thought menstruating women should avoid work for workplace hygiene would rather miss work, compared to 28% of those who did not agree with this belief. These findings suggest that while struggles related to managing menstrual bleeding may be less important for absenteeism, they are crucial for discomfort at work. Pain remained significantly associated with discomfort among the full sample, however this association was not significant in analyses including only women working in markets.

We found a statistically significant association between unmet menstrual health needs and women’s mental health. There are few studies against which to compare average WHO-5 scores for this sample, although scores suggested poor wellbeing. Mean scores observed in our study were poorer than those recorded for health care workers in Malawi [55] and similar to a sample of HIV-positive adults in Tanzania [56] as well as women in a study in India assessing the association between sanitation insecurity and mental health [57]. After adjustment for age and poverty, we found that unmet menstrual management needs, pain, and social support were all negatively associated with wellbeing in individual and multivariable comparisons. In contrast, reporting an attitude that menstruation should be kept secret, and that women should avoid work during menstruation for workplace hygiene were associated with better wellbeing in the full sample. While these findings may seem surprising when these attitudes were associated with absenteeism and a desire to avoid work, they are consistent with the findings from the qualitative interviews. In our qualitative analysis we found that women expressed pride in successfully enacting social expectations to keep menstruation secret and to keep clean [14]. It is plausible that for those endorsing and adhering to these expectations, this had a positive effect on wellbeing.

### Strengths and limitations

Survey questions and quantitative analyses were informed by in-depth qualitative investigation undertaken with the study population, along with past research. We also used our qualitative findings to aid the interpretation of results reported here, providing triangulation, and strengthening conclusions. While we were unable to take a full census and random sample of the workers, our proportional systematic sampling in markets offered a feasible and rigorous approach for this population. Only a small number of teachers and HCF workers were included due to feasibility constraints. These workers were included in the main quantitative analysis as similar consequences and unmet menstrual health needs were identified in the qualitative study and we hypothesised the same associations [14]. Quantitative sensitivity analysis including only women working in markets showed some differences to comparisons in the full sample. Future studies should investigate these effects in a larger sample of different worker groups. To assess menstrual management needs we used the newly validated Menstrual Practice Needs Scale to offer a comprehensive assessment [42,43]. We used the total score, rather than sub-scales to assess needs across the broad spectrum of blood management practices. However, this approach did include items capturing women’s experiences at home, not only those applicable to the workplace. Our findings are from cross-sectional data and as such we cannot infer causality or directionality. We elected to include a dichotomous variable indicating the presence of pain in multivariable analyses due to zero-inflation of pain reported on a rating scale. Questions capturing whether women were able to successfully reduce their menstrual pain should be explored in future studies to better understand the potential for interventions supporting pain mitigation. Our study relied on women’s self-reported unmet menstrual health needs and consequences for work. Such self-report is open to social desirability bias. Particularly in the interview format where women may have felt embarrassed to report difficulties surrounding menstruation to enumerators. Participants may have underreported unmet menstrual health needs or consequences of menstruation for their work.

We did not include any assessment of menstrual-related knowledge in our analysis. Inadequate knowledge about the menstrual cycle is often highlighted as a need among adolescents [58]. In our qualitative interviews, women did not report a high level of knowledge needs [14], although they desired more detailed information about menstrual products to inform their purchasing. Knowledge was not prioritized within the limited length of the quantitative survey.

### Implications for research and practice

Women spend many of their waking hours at work. In our sample, most worked six or seven days per week and between nine and 12 hours each day. Achieving sustainable development goals of decent work for all means ensuring work in safe environments, with equal opportunities for women. Our findings highlight that menstruation is an important contributor to women’s lives at work and must be considered in infrastructure provision and workplace policies [59,60].

Our findings demonstrate the importance of taking a holistic approach to menstrual health [4,7,12,16,61,62] which acknowledges the contribution of self-care challenges, pain, social support, attitudes, and norms. This range of drivers must be considered when designing programs to support women. We also found that different menstrual health needs may be more influential for some consequences. Comprehensive outcome assessment should be used in intervention trials and program evaluations to ensure the many consequences of menstruation for women’s lives are considered.

Menstrual pain was particularly important for work absenteeism, associated with a more than three-fold increase in missing work. Other correlates of absenteeism, being comfortable to talk to someone and expected to stay home during menstruation, may both serve to support women in managing their pain or symptoms at work. In contrast, unmet menstrual management needs were associated with a significantly increased desire to avoid work during menstruation, as did viewing menstruating women as dirty. Interventions focused on improving women’s access to infrastructure and materials may be more effective at reducing discomfort at work than absenteeism, while pain-focused interventions may be best placed to improve attendance. Use of improvised menstrual materials was associated with missing work, suggesting these may offer less protection against leakage despite women’s preferences. In contrast women’s own perspectives on their needs were associated with discomfort and wellbeing, while commercial product use was not.

Unmet menstrual health needs are associated with women’s wellbeing, with blood management needs and social support particularly important. At the same time, we found that attitudes constructing menstruation as dirty and something that should be kept secret had a positive association with wellbeing suggesting norm-change interventions must be navigated carefully.

## Supporting information

S1 File STROBE checklist. (PDF)

S2 File Sensitivity analyses including only women working in markets. (PDF)
