# Supplementary material for: Menstrual health challenges in the workplace and consequences for women’s work and wellbeing: A cross-sectional survey in Mukono, Uganda
Source: PLOS Glob Public Health. 2022 Jul 21;2(7):e0000589. doi: 10.1371/journal.pgph.0000589 (PMC10021399; doi:10.1371/journal.pgph.0000589)
Supplement: S1 File — (PDF) [file pgph.0000589.s001.pdf]

Supplementary Materials 1

STROBE Statement—Checklist of items that should be included in reports of ***cross-sectional studies***

	Item No	Recommendation	Page No
**Title and abstract**	1	(*a*) Indicate the study's design with a commonly used term in the title or the abstract	Title
		(*b*) Provide in the abstract an informative and balanced summary of what was done and what was found	Abstract
**Introduction**			
Background/rationale	2	Explain the scientific background and rationale for the investigation being reported	Introduction (pg. 4-5)
Objectives	3	State specific objectives, including any prespecified hypotheses	'The present study' pg 5.
**Methods**			
Study design	4	Present key elements of study design early in the paper	Abstract + Methods "Data collection"
Setting	5	Describe the setting, locations, and relevant dates, including periods of recruitment, exposure, follow-up, and data collection	Method, "Study Population" and "Data collection"
Participants	6	(*a*) Give the eligibility criteria, and the sources and methods of selection of participants	Method: "Study population"
Variables	7	Clearly define all outcomes, exposures, predictors, potential confounders, and effect modifiers. Give diagnostic criteria, if applicable	Method: "Measures"
Data sources/ measurement	8*	For each variable of interest, give sources of data and details of methods of assessment (measurement). Describe comparability of assessment methods if there is more than one group	Method: "Measures"
Bias	9	Describe any efforts to address potential sources of bias	Method: "Analysis"
Study size	10	Explain how the study size was arrived at	Method: "Study population" Paragraph 4
Quantitative variables	11	Explain how quantitative variables were handled in the analyses. If applicable, describe which groupings were chosen and why	Method: "Measures" & Analysis"
Statistical methods	12	(*a*) Describe all statistical methods, including those used to control for confounding	Method: "Analysis"
		(*b*) Describe any methods used to examine subgroups and interactions	Method: "Analysis" (supplementary materials 2)
		(*c*) Explain how missing data were addressed	Method: "Analysis"
		(*d*) If applicable, describe analytical methods taking account of sampling strategy	Method: "Analysis"
		(<u>*e*</u>) Describe any sensitivity analyses	Method: "Analysis"
**Results**			

Participants	13*	(a) Report numbers of individuals at each stage of study—eg numbers potentially eligible, examined for eligibility, confirmed eligible, included in the study, completing follow-up, and analysed	Results – "Respondents"
		(b) Give reasons for non-participation at each stage	NA
		(c) Consider use of a flow diagram	-
Descriptive data	14*	(a) Give characteristics of study participants (eg demographic, clinical, social) and information on exposures and potential confounders	Results – "Respondents" Table 1 "Sample Characteristics"
		(b) Indicate number of participants with missing data for each variable of interest	Results "Consequences for work and wellbeing" Table 2
Outcome data	15*	Report numbers of outcome events or summary measures	Table 2
Main results	16	(*a*) Give unadjusted estimates and, if applicable, confounder-adjusted estimates and their precision (eg, 95% confidence interval). Make clear which confounders were adjusted for and why they were included	"Contribution of menstrual health needs to consequences for work" Table 3, Table 4
		(*b*) Report category boundaries when continuous variables were categorized	Table 3, Table 4, Methods: "Measures"
		(*c*) If relevant, consider translating estimates of relative risk into absolute risk for a meaningful time period	Risk (Prevalence) ratios presented
Other analyses	17	Report other analyses done—eg analyses of subgroups and interactions, and sensitivity analyses	Supplementary Materials 2
**Discussion**			
Key results	18	Summarise key results with reference to study objectives	Discussion, paragraph 1-4
Limitations	19	Discuss limitations of the study, taking into account sources of potential bias or imprecision. Discuss both direction and magnitude of any potential bias	Discussion – "Strengths and Limitations"
Interpretation	20	Give a cautious overall interpretation of results considering objectives, limitations, multiplicity of analyses, results from similar studies, and other relevant evidence	Discussions – "Implications for research and practice"
Generalisability	21	Discuss the generalisability (external validity) of the study results	Discussion "Strengths and Limitations"
**Other information**			
Funding	22	Give the source of funding and the role of the funders for the present study and, if applicable, for the original study on which the present article is based	Funding statement and journal submission system.

*Give information separately for exposed and unexposed groups.

Note: An Explanation and Elaboration article discusses each checklist item and gives methodological background and published examples of transparent reporting. The STROBE checklist is best used in conjunction with this article (freely available on the Web sites of PLoS Medicine at http://www.plosmedicine.org/, Annals of Internal Medicine at http://www.annals.org/, and Epidemiology at http://www.epidem.com/). Information on the STROBE Initiative is available at www.strobe-statement.org.
