# Supplementary material for: Menstrual health challenges in the workplace and consequences for women’s work and wellbeing: A cross-sectional survey in Mukono, Uganda
Source: PLOS Glob Public Health. 2022 Jul 21;2(7):e0000589. doi: 10.1371/journal.pgph.0000589 (PMC10021399; doi:10.1371/journal.pgph.0000589)
Supplement: S2 File — (PDF) [file pgph.0000589.s002.pdf]

Supplementary Materials 2 – Sensitivity Analysis with participants working in markets.

MARKETS ONLY. Prevalence of menstrual health needs, bivariate and multivariable associations between menstrual health needs and work absenteeism and discomfort during menstruation.

	Total % (mean)	Missed work n (%) / M (SD)	Did not miss work n (%) / M (SD)	PR (95%CI)	aPR (95%CI)ǂ	Would prefer to miss work n (%)	Would not prefer to miss n (%)	PR (95%CI)	aPR (95%CI)ǂ
Poverty	(4.68)	5.67 (3.98)	4.47 (3.70)	1.06 (0.98-1.14)	-	5.17 (3.95)	4.27 (3.58)	**1.03 (1.01-1.05)**	0.99 (0.98-1.02)
**Managing menses**									
Menstrual Practice Needs total score (0-3)	(0.55)	0.67 (0.49)	0.52 (0.39)	**1.78 (1.19-2.66)**	1.26 (0.90-1.77)	0.63 (0.44)	0.48 (0.37)	**1.55 (1.28-1.87)**	**1.36 (1.17-1.59)**
Few unmet needs	54.7	36 (15.1)	202 (84.9)			98 (41.2)	14 (58.8)		
Some unmet needs	42.8	32 (17.3)	153 (82.7)			94 (50.6)	92 (49.5)		
Many unmet needs	2.5	4 (36.4)	7 (63.6)			7 (63.6)	4 (36.4)		
Uses improvised materials at work	23.6	24 (23.5)	78 (76.5)	**1.48 (1.16-1.89)**	**1.38 (1.05-1.80)**	55 (53.9)	47 (46.1)	1.21 (0.95-1.53)	
Uses commercial materials at work	76.4	47 (14.2)	284 (85.8)	1.00	1.00	143 (43.1)	189 (56.9)	1.00	
**Pain**									
Experiences pain	80.2	67 (19.3)	81 (94.2)	**2.92 (1.21-7.02))**	**2.88 (1.30-6.37)**	171 (49.0)	178 (51.0)	**1.47 (1.11-1.89)**	1.39 (0.99-1.94)
Does not experience pain	19.8	5 (5.8)	281 (80.8)	1.00	1.00	28 (32.6)	58 (67.4)	1.00	1.00
Pain severity (1-10)		5.92 (2.96)	4.41 (3.37)	**1.11 (1.07-1.15)**	-	5.27 (3.30)	4.14 (3.30)	**1.05 (1.02-1.09)**	-
**Social support at work**									
Comfortable talking to someone	45.2	26 (13.3)	170 (86.7)	1.00		82 (41.6)	115 (58.4)	1.00	1.00
Not comfortable talking to someone	54.8	46 (19.3)	192 (80.7)	**1.40 (1.06-1.85)**	**1.49 (1.02-2.17)**	117 (49.2)	50.84 (121)	1.18 (0.98-1.41)	**1.15 (1.02-1.30)**
Has someone she could ask for help	40.8	34 (19.2)	143 (80.8)	1.00	-	81 (45.5)	97 (54.5)	1.00	-
Does not have someone	59.2	38 (14.8)	219 (85.2)	0.79 (0.53-1.15)	-	118 (45.9)	139 (54.1)	1.01 (0.88-1.15)	-
**Sociocultural environment: Attitudes & norms**									
Attitude: Menstruation should be kept secret									
Agree	64.4	48 (17.2)	231 (82.8)	1.09 (0.60-1.98)	-	134 (48.0)	145 (52.0)	1.14 (0.97-1.33)	0.98 (0.86-1.11)
Disagree	35.6	24 (15.6)	130 (84.4)	1.00	-	65 (41.9)	90 (58.1)	1.00	1.00
Attitude: Women should avoid work during menstruation for hygiene									
Agree	36.3	39 (24.8)	118 (75.2)	**1.95 (1.36-2.80)**	1.40 (0.88-2.23)	106 (67.1)	52 (32.9)	**1.98 (1.67-2.34)**	**1.71 (1.44-2.02)**
Disagree	63.7	33 (12.0)	242 (88.0)	1.00	1.00	92 (33.5)	183 (66.6)	1.00	1.00
Injunctive norm: Women are expected to keep menstruation secret									
Agree	75.6	58 (17.9)	267 (82.2)	**1.45 (0.97-2.16)**	1.04 (0.71-1.53)	154 (47.2)	172 (52.8)	1.21 (0.89-1.64)	-
Disagree	24.4	23 (12.4)	92 (87.6)	1.00	1.00	41 (39.1)	64 (61.0)	1.00	
Injunctive norm: Women are expected to stay home when menstruating									
Agree	17.9	28 (36.4)	49 (63.6)	**2.88 (2.10-3.95)**	**2.49 (1.59-3.88)**	55 (70.5)	23 (29.5)	**1.75 (1.44-2.14)**	**1.39 (1.09-1.78)**
Disagree	82.1	42 (11.9)	312 (88.1)	1.00	1.00	141 (39.8)	213 (60.2)	1.00	1.00
Most shoppers would avoid purchasing food from a menstruating women									
Agree	60.1	51 (19.7)	208 (80.3)	**1.50 (1.04-2.19)**	1.17 (0.77-1.77)	140 (53.9)	120 (46.1)	1.60 (0.99-2.58)	**1.39 (1.07-1.80)**
Disagree	39.9	21 (12.2)	151 (87.8)	1.00	1.00	57 (33.1)	115 (66.9)	1.00	1.00

PR: Prevalence Ratio. aPR: Adjusted prevalence ratio. CI: Confidence Interval. ǂ Confidence intervals using Kauermann-Carroll correction were extreme due to the very small number of clusters (n=10), noted as problematic elsewhere,[1] thus CI's reported do not adjust for the small number of clusters

1. Thompson J, Hemming K, Forbes A, et al. Comparison of small-sample standard-error corrections for generalised estimating equations in stepped wedge cluster randomised trials with a binary outcome: A simulation study. *Statistical methods in medical research* 2021;30(2):425-39.

MARKETS ONLY. Associations between reported menstrual health needs and wellbeing measured using the WHO-5

	**Model 1 (predictor with adjustment for age & poverty)**		**Model 2 (full multivariable model)**	
**Predictor**	**b (Std. Error)**	**95%CI**	**b (Std. Error)**	**95%CI**
Age	**-0.50 (0.11)**	-0.75, -0.24	**-0.41 (0.14)**	-0.72, -0.09
Poverty	**-1.98 (0.25)**	-2.55, -1.41	**-1.73 (0.27)**	-2.33, -1.12
**Managing menses**				
Menstrual Practice Needs total score	**-7.67 (1.41)**	-10.85, -4.48	**-6.55 (1.45)**	-9.84, -3.26
Uses improvised materials at work (yes)	2.66 (1.53)	-0.80, 6.13	-	
**Pain**				
Experiences pain (yes)	**-3.63 (2.13)**	-8.44, 1.19	**-**	
Pain severity (1-10)	-0.52 (0.31)	-1.23, 0.18	-	
**Social support at work**				
Comfortable talking to someone (No)	**-4.77 (1.68)**	-8.57, -0.98	**-5.22 (1.99)**	-9.72, -0.71
Has someone she could ask for help (No)	-0.29 (2.00)	-4.83, 4.25	-	
**Attitudes & norms**				
Attitude: Menstruation should be kept secret (agree)	3.95 (2.01)	-0.60, 8.50	5.00 (2.40)	-0.43, 10.42
Attitude: Women should avoid work during menstruation for hygiene (agree)	**4.81 (2.05)**	0.18, 9.44	4.54 (2.39)	-0.86, 9.95
Injunctive norm: Women are expected to keep menstruation secret (agree)	-0.25 (2.18)	-5.18, 4.68	-	
Injunctive norm: Women are expected to stay home when menstruating (agree)	**4.35 (1.39)**	1.21, 7.50	2.37 (1.49)	-0.86, 9.95
Most shoppers would avoid purchasing food from a menstruating woman	-0.18 (2.25)	-5.26, 4.91	-	
Intercept			68.58 (1.49)	
Adj R^2			0.18	

b: regression coefficient. Std. Error: Robust standard error with cluster adjustment.
